# Coordinate Systems for Navigating Stereotactic Space: How Not to Get Lost

**DOI:** 10.7759/cureus.8578

**Published:** 2020-06-12

**Authors:** Mark Sedrak, Armando L Alaminos-Bouza, Siddharth Srivastava

**Affiliations:** 1 Neurosurgery, Northern California Kaiser Permanente, Redwood City, USA; 2 Medical Physics, MEVIS Informática Médica Ltda., São Paulo, BRA; 3 Neuroscience, Northern California Kaiser Permanente, Redwood City, USA

**Keywords:** cartesian coordinate system, euclidean space, stereotactic and functional, coordinate transformation, stereotactic frame

## Abstract

All stereotactic neurosurgical procedures utilize coordinate systems to allow navigation through the brain to a target. During the surgical planning, indirect and direct targeting determines the planned target point and trajectory. This targeting allows a surgeon to precisely reach points along the trajectory while minimizing risks to critical structures. Oftentimes, once a target point and a trajectory are determined, a frame-based coordinate system is used for the actual procedure. Considerations include the use of various coordinate spaces such as the anatomical (${X}$), the frame (${X'}$), the head-stage (${X''}$), and an atlas. Therefore, the relationships between these coordinate systems are integral to the planning and implementation of the neurosurgical procedure. Although coordinate transformations are handled in planning via stereotactic software, critical understanding of the mathematics is required as it has implications during surgery. Further, intraoperative applications of these coordinate conversions, such as for surgical navigation from the head-stage, are not readily available in real-time. Herein, we discuss how to navigate these coordinate systems and provide implementations of the techniques with samples.

## Introduction

In 1908, Sir Victor Horsley (1857-1916), a neurosurgeon, and Robert Clarke (1850-1926), a physiologist, ignited the field of stereotactic neurosurgery by introducing a frame to navigate the structures of the *Macacus rhesus* cerebellum methodically using "electrolytic" lesions [1, 2]. By 1947, Ernest Spiegel (1895-1985), a neurologist, and Henry Wycis (1911-1972), a neurosurgeon, applied these frame techniques for human use in the treatment of pain, epilepsy, mental disorders, movement disorders, and tumors [3-5]. While at that time the devices were primitive, the idea was revolutionary as it was built on mathematics applied to an apparatus designed to navigate regions of the brain. In 1978, the next big leap forward was an invention by Russell Brown that precisely mapped computed tomography (CT) imaging with a stereotactic frame using an N-localizer [6-8]. This creation, which later merged with image fusion and magnetic resonance imaging (MRI), allowed precise stereotactic targeting in neurosurgery.

Historically, coordinate systems are a key element in the practice of stereotactic neurosurgery. These systems are utilized during the surgical procedures; therefore, a robust understanding is critical for those using them. For this reason, we hope to re-introduce some important concepts previously published and expand upon them further as a complete solution for the surgical technique.

## Technical report

Background transformations

Various Cartesian coordinate systems in Euclidean space are utilized in stereotactic neurosurgery. The affine conversion of one coordinate system to another is often computed from one coordinate set ${X'_{xyz}}$ to the other coordinate set ${X_{xyz}}$ using matrices that specifies information on rotation ${R'}$, scaling ${S'}$, and translation ${T'}$ (Equation 1). These conversion matrices can be solved using three or more points using various methods [9-11]. The components of ${X_{xyz}}$ and ${X'_{xyz}}$ consists of $[{x, y, z}]^{T}$ and $[{x', y', z'}]^{T}$, respectively. The rotational matrix consists of nine components $r$, while the scaling $s$ and translation $t$ components have three (Equations 2). It should also be noted that the matrix components can also be treated as individual functions in a non-linear fashion. For example, scaling factors can be functions ($f(s_{x'})$, $f(s_{y'})$ or $f(s_{z'})$) used to compensate for distortions of the brain relative to an atlas.

$$X_{xyz} = R' \cdot S' \cdot X'_{xyz} + T' \tag{1}$$

$$\begin{bmatrix} x \\ y \\ z \end{bmatrix} = \begin{bmatrix} r_{x'x} & r_{x'y} & r_{x'z} \\ r_{y'x} & r_{y'y} & r_{y'z} \\ r_{z'x} & r_{z'y} & r_{z'z} \end{bmatrix} \cdot \begin{bmatrix} s_{x'} & 0 & 0 \\ 0 & s_{y'} & 0 \\ 0 & 0 & s_{z'} \end{bmatrix} \cdot \begin{bmatrix} x' \\ y' \\ z' \end{bmatrix} + \begin{bmatrix} t_{x'} \\ t_{y'} \\ t_{z'} \end{bmatrix}\tag{2}$$

**Figure 1:**
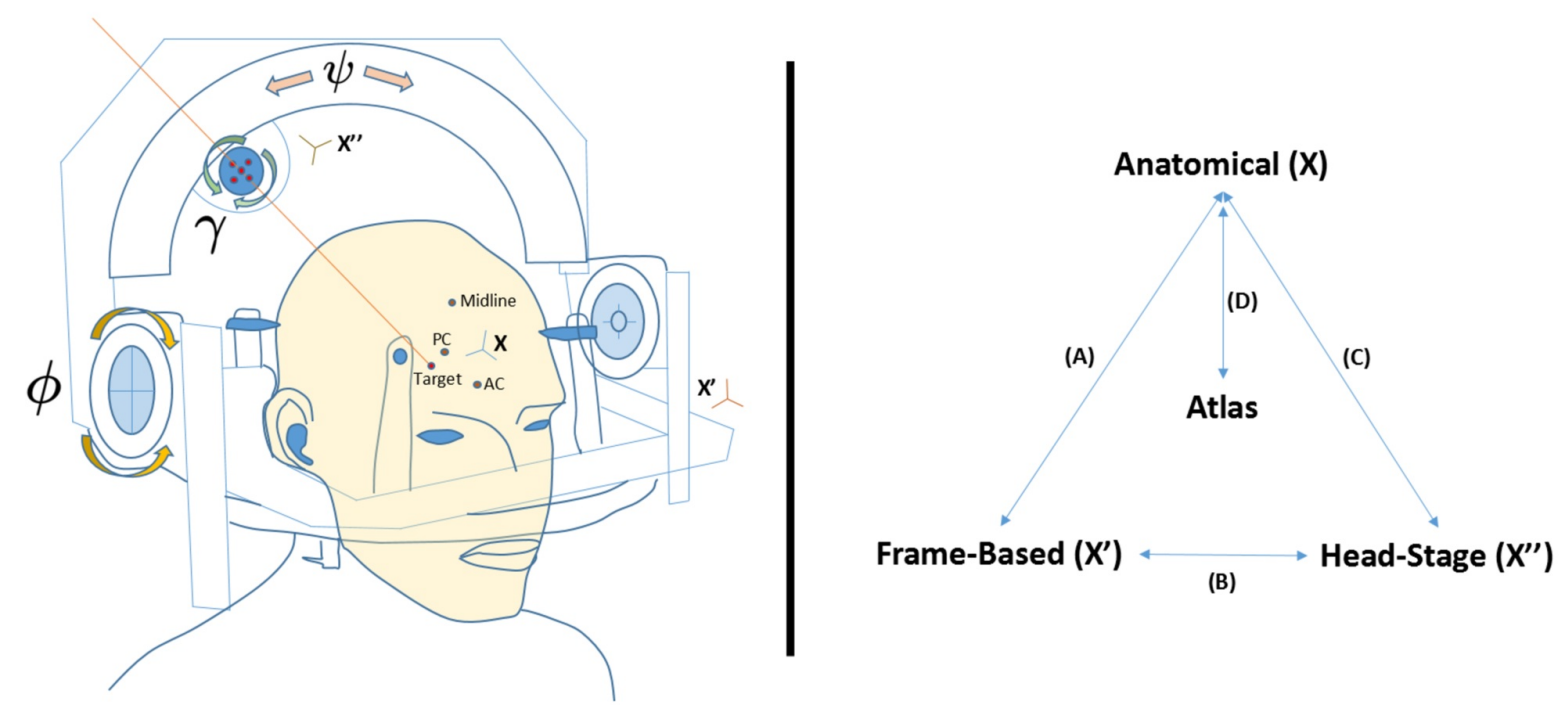
Frame-based stereotaxis involves several coordinate spaces, which include the anatomical space ${X}$, the frame space ${X'}$, and the head-stage space ${X''}$. These spaces are defined in surgery once a frame is attached to a patient and has undergone stereotactic localization (Left). Then, one can perform coordinate transformations between all three systems and the anatomical space to an atlas for which the mathematical computations are referenced in later sections of the article (Right, A-D). Conversion D can also be utilized with computed tomography (CT) or magnetic resonance imaging (MRI) rather than an atlas. Important reference points include the anterior commissure (AC), posterior commissure (PC), and the midline. In frames that operate around an isocenter, which are known as target-centered frames, the head-stage rotates around the frame-space. Three angles can be applied to the head-stage, which are in frame space the ring angle $\phi$, the arc angle $\psi$, and the axial angle $\gamma$ representing rotations about the x-axis, the y-axis, and the z-axis, respectively. In anatomical space, $\phi$ represents the sagittal angle, whereas $\psi$ represents the coronal angle. The $\gamma$ angle can also be used for electrode rotation. In addition, there are movements in the head-stage along the AP, LAT, and Vertical (depth) axes. AP = Antero-Posterior; LAT = Lateral; VERT = Vertical; X = Anatomical space; X' = Frame-based space; X'' = Head-stage space; AC = Anterior Commissure; PC = Posterior Commissure; Midline = Midline structure $\phi \: = \: x-axis\: rotation$ $\psi \: = \: y-axis\: rotation$ $\gamma \: = \: z-axis \: rotation$ Left Image Pane. Depiction of the Cosman-Roberts-Wells (CRW) Stereotactic Apparatus (Radionics CRW Stereotactic System, Integra LifeSciences Corporation, Plainsboro, New Jersey)

The constitution of the component matrices (Equation 2), which allow conversion from one coordinate to another, depends on the space for which the mathematical operation is being performed. Some of these coordinate spaces include an anatomical space ${X}$, a frame-based space ${X'}$, and a head-stage space ${X''}$ (Figure 1). Frequently, an atlas is used in reference to the anatomical space. The general scheme of Equation 1 can be applied in the transformations of these coordinate spaces for which each operation is discussed in detail (Figure 1, Right Pane, A-D) in subsequent sections. Of note, the x-axis is generally considered the left-right (LAT) direction, the y-axis is the back-front (AP) direction, and finally the z-axis the down-up (VERT) direction, which is right-anterior-superior (RAS) convention; however, some conventions flip the x-y axes making the x-axis AP and y-axis LAT. Lastly, anatomical space is built off of reference points in the brain, such as the anterior commissure (AC), posterior commissure (PC), and a midline point (Midline), whereas frame-based space is generated using an N-localizer [6-8]. We also discuss head-stage to anatomical transformation in frameless stereotaxy in addition to computation of coordinates along a fixed trajectory, both of which are generally in anatomical space.

Anatomy-Frame Transformation (A)

A method of rigid coordinate transformation in stereotactic neurosurgery without the need for scaling because the systems are all in units of millimeters (mm), using three points has been previously published, but is worth reviewing [12-15]. In this 3-point transformation (3PT), one can think of the problem in matrix form where ${R'}_{i'i}$ represents an unknown rotational matrix from frame-to-anatomic systems, ${X}$ represents the anatomic coordinate space, ${X}'$ is the frame coordinate space, and $P_{M}'$ represents a translation (Equation 3). The unknowns are ${R'}_{i'i}$, which represent the unknown elements of the rotational matrix, and $P_{M}'$, which represents a translation (Equation 4). Likewise, the reverse coordinate transformation is possible (Equations 5, 6), where (-1) represents the matrix inverse.

$$X = {R'}_{i'i} \cdot (X' - P_{M}') \tag{3}$$

$$\begin{bmatrix} x_{1}\\ y_{2}\\ z_{3} \end{bmatrix} = \begin{bmatrix} \hat{i_{x}'}\cdot \hat{i_{x}} & \hat{i_{x}'}\cdot \hat{i_{y}} & \hat{i_{x}'}\cdot \hat{i_{z}}\\ \hat{i_{y}}'\cdot \hat{i_{x}} & \hat{i_{y}'}\cdot \hat{i_{y}} & \hat{i_{y}'}\cdot \hat{i_{z}}\\ \hat{i_{z}'}\cdot \hat{i_{x}} & \hat{i_{z}'}\cdot \hat{i_{y}} & \hat{i_{z}'}\cdot \hat{i_{z}} \end{bmatrix} \cdot \begin{bmatrix} \begin{bmatrix} x_{1}'\\ y_{2}'\\ z_{3}' \end{bmatrix} - \begin{bmatrix} p_{m1}'\\ p_{m2}'\\ p_{m3}' \end{bmatrix} \end{bmatrix}\tag{4}$$

$$X' = {R'}_{i'i}^{-1} \cdot X + P_{M}' \tag{5}$$

$$\begin{bmatrix} x_{1}'\\ y_{2}'\\ z_{3}' \end{bmatrix} = \begin{bmatrix} \hat{i_{x}'}\cdot \hat{i_{x}} & \hat{i_{x}'}\cdot \hat{i_{y}} & \hat{i_{x}'}\cdot \hat{i_{z}}\\ \hat{i_{y}}'\cdot \hat{i_{x}} & \hat{i_{y}'}\cdot \hat{i_{y}} & \hat{i_{y}'}\cdot \hat{i_{z}}\\ \hat{i_{z}'}\cdot \hat{i_{x}} & \hat{i_{z}'}\cdot \hat{i_{y}} & \hat{i_{z}'}\cdot \hat{i_{z}} \end{bmatrix}^{-1} \cdot \begin{bmatrix} x_{1}\\ y_{2}\\ z_{3} \end{bmatrix} + \begin{bmatrix} p_{m1}'\\ p_{m2}'\\ p_{m3}' \end{bmatrix}\tag{6}$$

Now we identify the components in more detail. First, we consider the stereotactic space relative to the middle of the anterior commissure (AC, $P_{AC}$ ) and posterior commissure (PC, $P_{PC}$ ), which we refer to as $midACPC$ but is alternatively termed as the mid-commissural coordinate system. At this point, the $midACPC$ space $X$ is 0 in the antero-posterior (AP), 0 in the lateral (LAT), and 0 in the vertical (VERT) ($P_{M}$ = {0,0,0}). This same point, however, exists in stereotactic frame-space $X'$. The $midACPC$ point in frame-space $P_{M}'$ will generally never be {0,0,0}, but rather some other values, by virtue of frame-based coordinates design or misalignment of the two systems during frame placement.

To facilitate subsequent transformation, it is now useful to obtain three points of reference in frame-based space. Generally, these points consist of the AC ( $P_{AC}'$ ) , PC ( $P_{PC}'$ ), and a midpoint ( $P_{F}'$ ) above or below a line connecting AC to PC, but not along that line; for instance, the falx cerebri or a midline ventricular structure are good locations to obtain $P_{F}'$. Using $P_{AC}'$ and $P_{PC}'$, we can now compute the $P_{M}'$ by taking their simple average (Equation 7).

$$P_{M}' = (P_{AC}' + P_{PC}')/2 \tag{7}$$

To complete the conversion, we must now determine the components of the rotational matrix ${R'}_{i'i}$. In $midACPC$ space, the basis unit vector is defined as (1,0,0), (0,1,0), and (0,0,1), which are $\hat{i_{x}}$, $\hat{i_{y}}$, $\hat{i_{z}}$ respectively. In frame-based space, two vectors, $\vec{v_{1}'}$ and $\vec{v_{2}'}$, are created by using the three points, $P_{AC}'$, $P_{M}'$, and $P_{F}'$, which will allow us to compute the unit vectors $\hat{i_{x}'}$, $\hat{i_{y}'}$, $\hat{i_{z}'}$ (Equations 8, 9).

$$\vec{v_{1}'} = P_{AC}' -P_{M}' \tag{8}$$

$$\vec{v_{2}'} = P_{F}' -P_{M}' \tag{9}$$

Using the two vectors $\vec{v_{1}'}$ and $\vec{v_{2}'}$, we can next convert these into unit vectors $\hat{v_{1}'}$ and $\hat{v_{2}'}$, by dividing them by their own magnitude (Equations 10, 11).

$$\hat{v_{1}'} = \vec{v_{1}'} / \parallel {\vec{v_{1}'}} \parallel \tag {10}$$

$$\hat{v_{2}'} = \vec{v_{2}'} / \parallel {\vec{v_{2}'}} \parallel \tag {11}$$

The unit vector $\hat{i_{y}'}$ is equivalent to $\hat{v_{1}'}$. The result of the cross-product of $\hat{v_{1}'} \times \hat{v_{2}'}$ gives $\hat{i_{x}'}$, when divided by the resulting magnitude. Finally, the result of the cross-product $\hat{i_{x}'} \times \hat{i_{y}'}$ gives $\hat{i_{z}'}$, when divided by the resulting magnitude. These cross-products follow the right-hand rule convention and if the midpoint ( $P_{F}'$ ) is below the AC-PC line, then the cross-product for $\hat{i_{x}'}$ would be $\hat{v_{2}'} \times \hat{v_{1}'}$. Next, the dot-products of the unit vectors between the coordinate systems give us the components of the matrix ${R'}_{i'i}$ (Equation 12).

$${R'}_{i'i} = \begin{bmatrix} \hat{i_{x}'}\cdot \hat{i_{x}} & \hat{i_{x}'}\cdot \hat{i_{y}} & \hat{i_{x}'}\cdot \hat{i_{z}}\\ \hat{i_{y}}'\cdot \hat{i_{x}} & \hat{i_{y}'}\cdot \hat{i_{y}} & \hat{i_{y}'}\cdot \hat{i_{z}}\\ \hat{i_{z}'}\cdot \hat{i_{x}} & \hat{i_{z}'}\cdot \hat{i_{y}} & \hat{i_{z}'}\cdot \hat{i_{z}} \end{bmatrix}\tag{12}$$

Head-Stage-Frame Transformation (B)

Whereas the target is defined by a point, the trajectory through the brain to a target is defined by a direction and a probe-insertion depth. In frame-based stereotaxis, the surgical space has a coordinate basis related to the surgical head-stage $X''$ for which the trajectory angles and the electrode/probe depth need to be calculated. This important coordinate system is directly utilized by the surgeon in real-time after the anatomic and frame-based systems are set. We consider here isocentric frame-based systems, which allow rotations around a target (target-centered), but maintain the same radial distance to target. In most frame systems, there is an arc angle $\psi$, a ring angle $\phi$, an "XY Stage", and a depth. The target and frame angles are usually predetermined in planning software, but may be slightly adjusted in real-time to accommodate brain structures, the burr hole, and physiology while the depth is determined manually using fine vernier scales or digitally using microdrives. Generally, the transformation matrix $R_{xy}$ comprises two angles, where $\phi$ is an angle about the x-axis $R_{x}$ (Equation 13) and $\psi$ is the angle about the y-axis $R_{y}$ (Equation 14). In some systems, it may be possible to have a rotational matrix $R_{z}$ about the z-axis $\gamma$, which can also be used for electrode rotation (Equation 15). $R_{xy}$ is therefore a combined rotational matrix corresponding to a rotation of $\psi$ about the AP axis, and a rotation of $\phi$ about the LAT axis, and no rotations about the VERT axis (Equation 16). A combination of all three rotational angles into a single matrix $R_{xyz}$ may also be utilized in some systems (Equation 17). Using this transformation, a movement in the direction of AP, LAT, or VERT on the head-stage $\Delta_{X''}$ is then converted to a new target frame-based point $X'_{n}$ using the current frame-based point $X'_{c}$ and $R'_{xy}$, which has combined rotation angles in the frame-based system (Equation 18). The conversion from $X'$ to $X$ is the same as previously mentioned. Finally, to obtain $\Delta_{X''}$, one can utilize the current frame-based point $X'_{c}$, a desired new target frame-based point $X'_{t}$, ${R'}_{xy}^{-1}$, and rearrange the terms of Equation 18 (Equation 19). Because ${R'}_{xy}^{-1}$ is a combined Euler rotational matrix, it is equivalent to the transpose of $R'_{xy}$.

$$ R_{x} = \begin{bmatrix} 1 & 0 & 0 \\ 0 & cos(\phi) & sin(\phi) \\ 0 & -sin(\phi) & cos(\phi) \end{bmatrix} \tag{13}$$

$$ R_{y} = \begin{bmatrix} cos(\psi) & 0 & sin(\psi) \\ 0 & 1 & 0 \\ -sin(\psi) & 0 & cos(\psi) \end{bmatrix} \tag{14}$$

$$ R_{z} = \begin{bmatrix} cos(\gamma) & sin(\gamma) & 0 \\ -sin(\gamma) & cos(\gamma)& 0 \\ 0 & 0 & 1 \end{bmatrix} \tag{15}$$

$$R_{xy} = R_{x} \cdot R_{y} \tag{16}$$

$$R_{xyz} = R_{x} \cdot R_{y} \cdot R_{z}\tag{17}$$

$$X'_{n} = X'_{c} + R'_{xy} \cdot \Delta_{X''} \tag {18}$$

$$\Delta_{X''} = {R'}_{xy}^{-1} \cdot (X'_{t} - X'_{c}) \tag {19}$$

It should be noted here that different frame systems have different displacement and angular components. For example, in CRW (Radionics CRW Stereotactic System, Integra LifeSciences Corporation, Plainsboro, New Jersey), LAT to the right is (+), AP towards anterior is (+), and VERT upwards is (+). In Leksell G (Elekta, Stockholm, Sweden), however, LAT to the right is (-), AP anterior is (+), and VERT upwards is (-) (Table 1). It is notable that angular data can also be used in anatomic space and one can also convert to a spherical coordinate system. Further, many of the angles in the systems may be offset from $\frac{\pi}{2}$, such as the ring angle in CRW. Also, in CRW, when the $\psi$ is to the right, the angle is positive, but when it is to the left, it is negative; when $\phi$ is anterior, the angle is positive, but when it is posterior, it is negative. Another issue is the apparatus assembly, which would flip these angles. Therefore, numerical data for angles in each system require a modification prior to use. Lastly, some frame systems are not target-centered ("isocentric") and other considerations are required.

**Table 1:** Direction of displacements changes by target-centered frame-based coordinate system. MidACPC coordinate space axes are similar to CRW, Micromar, FiME, Macom, Zeppelin. However, Leksell G has flipped signs of displacement on the LAT and VERT directions, where the origin is right-posterior-superior. The CRW and Leksell G base rings are fixed inferiorly, whereas the others can be fixed additionally in a more superior position. Lastly, N-localizer dimensions are compared. *Larger N-localizer for radiosurgery N-localizer - see references [6-8] mm = millimeters; AP = Antero-Posterior; LAT = Lateral; VERT = Vertical; H x W = Height and Width; MidACPC = Middle of anterior commissure and posterior commissure CRW = Cosman-Roberts-Wells stereotactic frame, Radionics CRW Stereotactic System, Integra LifeSciences Corporation, Plainsboro, NJ, USA Leksell G = Leksell Stereotactic Frame, Elekta, Stockholm, Sweden Micromar Ind. Com. Ltda, Diadema, Sao Paulo, Brazil FiMe Medica SRL, Cordoba, Argentina Macom Instrumental Cirurgico, Sao Paulo, Brazil Zeppelin Adeor Medical AG, Martinshof 5, D-83626 Valley, Germany

	Offset						
	Origin-center	Origin-center	Origin-base				N-Localizer
Stereotactic Frame	LAT (mm)	AP (mm)	VERT (mm)	Right	Anterior	Superior	[H x W] (mm)
CRW	0	0	-86	+	+	+	189 x 140
Leksell G	100	100	160	-	+	-	120 x 120
Micromar	0	0	0	+	+	+	140 x 140 & 175 x 140*
FiMe	0	0	0	+	+	+	160 x 160 & 200 x 160*
Macom	0	0	0	+	+	+	140 x 140
Zeppelin	0	0	0	+	+	+	140 x 140

Head-Stage-Anatomy Transformation in Frame-Based Stereotaxy (C)

Multiplying the matrix ${R'}_{i'i}$ by the matrix ${R'}_{xy}$ produces the matrix $W'$ that transforms coordinates from the head-stage to $midACPC$ (Equation 20). The complete transformation from a movement on the head-stage $\Delta_{X''}$ to a new anatomic point $X_{n}$ utilizes the current $midACPC$ point $X_{c}$, the $\Delta_{X''}$, and $W'$ (Equation 21). Finally, to move to a desired $midACPC$ target point $X_{t}$, the head-stage movements can be computed (Equation 22).

$$W' = {R'}_{i'i}\cdot {R'}_{xy} \tag {20}$$

$$X_{n} = X_{c} + W' \cdot \Delta_{X''} \tag {21}$$

$$\Delta_{X''} = {W'}^{-1} \cdot (X_{t}-X_{c}) \tag {22}$$

Head-Stage-Anatomy Transformation in Frameless Stereotaxy

In some systems, frameless stereotaxy is utilized. In these instances, there is a single anatomical coordinate system, $midACPC$. However, the head-stages may have calibrated displacements $\Delta_{X''}$ in addition to the optical tracking systems. As some of the systems use all three angles, a combined anatomical rotational matrix $R_{xyz}$ can be utilized, which incorporates a coronal angle $\psi$, a sagittal angle $\phi$, and an axial angle $\gamma$, which are different than the frame-based angles previously mentioned (Equation 17). The complete transformation from a movement on the head-stage $\Delta_{X''}$ to a new anatomic point $X_{n}$, utilizes the current $midACPC$ point $X_{c}$, the $\Delta_{X''}$, and $R_{xyz}$ (Equation 23). Finally, to move to a desired $midACPC$ target point $X_{t}$, the head-stage movements can be computed (Equation 24).

$$X_{n} = X_{c} + R_{xyz} \cdot \Delta_{X''} \tag {23}$$

$$\Delta_{X''} = R_{xyz}^{-1} \cdot (X_{t}-X_{c}) \tag {24}$$

Calculation of Points Along a Single Fixed Trajectory

While the above sections provide insight into full computations from head-stage to anatomy, it may be useful to compute points along a single fixed trajectory. For this method, two points are required, an entry $P_{e}$ and a target $P_{t}$. Also required, the third element is the displacement along the trajectory, which can be treated as a fraction $f _{et}$ of the vector $P_{e}$ to $P_{t}$. This fraction is easily derived in stereotactic neurosurgery from the millimeter distances on vernier scales or computerized microdrives from entry to target as the current scalar distance $d_{c}$ for the numerator, while the denominator is the Euclidean distance $d_{et}$ from $P_{e}$ to $P_{t}$ (Equation 25). Finally, a new position $P_{i}$ can be computed (Equation 26).

$$f_{et} = d_{c}/d_{et} \tag{25}$$

$$P_{i} = P_{e} + f_{et} *(P_{t} - P_{e}) \tag{26}$$

Implementation

The following implementation of the discussed mathematics were computed in a Stereotactic Calculator (STACC) and other tools using custom software available at https://app.box.com/s/4zxysoc3bltxvvt4cwcplpbkntoi9cll. A complete plan was utilized from iPlan Stereotaxy 3.0 (Brainlab, Inc., Feldkirchen, Germany) with all the essential coordinates from $midACPC$ and CRW (Table 2). In this computation, several points are gathered in CRW and $midACPC$ space in order to perform the conversions. A comparison between the coordinate conversion using 3PT versus using 5-points was made. The 5-point conversion utilized a least-squares computation between five pairs of coordinates [9]. Comparing the result with the iPlan target values demonstrates a very slight improvement in the Euclidean error rates when using the 5-point technique as compared to the 3PT. This is likely due to a decimal point round-off error as provided by the planning station.

**Table 2:** CRW and MidACPC coordinates obtained from iPlan stereotactic planning software. The Euclidean error in computation of the target point as compared to the point provided by iPlan using 3-points are 0.13 mm and 0.06 mm on the right and left, respectively. The Euclidean error using a 5-point least-squares computation for targets as compared to iPlan are 0.042 mm on both sides. The Euclidean distance from AC to PC was 24.45 mm and half of the value was 12.23 mm, which matched the value provided by the planning system in MidACPC space. Of note, the CRW ring angle is offset by 90 degrees. iPlan Stereotaxy 3.0 (Brainlab, Inc., Feldkirchen, Germany) MidACPC = Middle of Anterior Commissure and Posterior Commissure; CRW = Cosman-Roberts-Wells; AP = Antero-Posterior; LAT = Lateral; VERT = Vertical; STN = Subthalamic Nucleus; AC = Anterior Commissure; PC = Posterior Commissure.

CRW	AP	LAT	VERT	
AC CRW	14.2	2.9	2.4	
PC CRW	-10.1	2.1	5	
Mid-Falx CRW	9.3	5.9	54.2	
Right CRW Target from Planning Station	-1.8	13.6	0.3	
Left CRW Target from Planning System	-0.8	-9.3	1.7	
Right CRW Target from Planning Station	-1.8	13.6	0.3	
Left CRW Target from Planning System	-0.8	-9.3	1.7	
MidACPC	AP	LAT	VERT	
AC MidACPC	12.23	0	0	
PC MidACPC	-12.23	0	0	
Mid-Falx MidACPC	1.98	0	51.09	
Right STN Target MidACPC	-3	11.5	-3	
Left STN Target	-3	-11.5	-3	
Computed Data - 3 Points	AP	LAT	VERT	
Right CRW Computed Target	-1.69673	13.67524	0.334593	
Left CRW Computed Target	-0.79024	-9.26333	1.748831	
Computed Data - 5 Points	AP	LAT	VERT	
Right CRW Computed Target	-1.77279	13.62566	0.318858	
Left CRW Computed Target	-0.77279	-9.27434	1.718858	
CRW Angles	Ring Angle	Arc Angle	Coronal/Lat Angle	Sagittal/AP Angle
Right	51.8	20.5	20.5	38.2
Left	51.6	10.1	-10.1	38.4
MidACPC Angles	Coronal/LAT Angle	Sagittal/AP Angle		
Right	19.0642	32.02104		
Left	-16.7686	32.44535		

Next, we analyzed the matrix used to compute the conversion between $midACPC$ and CRW. The resultant matrices in the forward and reverse match between 3PT and 5-point methods (Table 3). Further, we analyzed the orthonormality of each 1 x 3 vector component, with the expectation that the quantity would be 1. Orthogonality compares the vector components between the first and second rows, the second and third rows, and the first and third rows. In an orthogonal system, one would expect the result to be 0. In Table 1, we can see that we have good normality and orthogonality with all the matrices.

**Table 3:** Comparison of resultant matrices computed from 3-point change of basis technique and 5-point linear least squares method. The matrices are presented from CRW-to-MidACPC and the reverse as 3 x 4, where the 3 x 3 components are the rotational vectors and the last column is the translation. One can observe that the matrices are very similar and a simple delta, subtraction, between each is presented. Analysis of the 3 x 3 vector component of each matrix by orthonormality and orthogonality are close to 1 and 0, respectively, which would be expected in this affine system. CRW = Cosman-Roberts-Wells; Mid-ACPC = Middle of Anterior Commissure (AC) and Posterior Commissure (PC); AP = Antero-Posterior; LAT = Lateral; VERT = Vertical.

CRW-					MidACPC-				
MidACPC	Matrix 1:				CRW	Matrix 2:			
Change of Basis	0.993792	-0.03941	0.104036	2.05		0.993792	0.032717	-0.106	-1.726
3-points	0.032717	0.997329	0.065298	2.5		-0.03941	0.997329	-0.061	-2.185
	-0.10633	-0.06149	0.992428	3.7		0.104036	0.065298	0.9924	-4.049
	Matrix 3:					Matrix 4:			
Linear Least Squares	0.993983	-0.04348	0.104011	2.021192		0.993471	0.036872	-0.107	-1.707
5-points	0.033201	0.995652	0.065853	2.472828		-0.03998	0.998827	-0.062	-2.161
	-0.10593	-0.06087	0.992991	3.680035		0.103533	0.065161	0.9919	-4.021
Delta	-0.00019	0.004065	2.51E-05	0.028808		0.000322	-0.00415	0.0002	-0.018
	-0.00048	0.001677	-0.00056	0.027172		0.000563	-0.0015	0.0006	-0.024
	-0.0004	-0.00062	-0.00056	0.019965		0.000503	0.000137	0.0005	-0.028
		Orthonormality	Orthonormality	Orthonormality	Orthogonality	Orthogonality	Orthogonality		
		AP	LAT	VERT	AP-LAT	LAT-VERT	AP-VERT		
	Matrix 1	1	1	1	-1.39E-15	1.19E-15	3.25E-15		
	Matrix 2	1	1	1	1.26E-15	-1.33E-15	-2.73E-15		
	Matrix 3	1.000711	0.996762	1.000959	-0.00344	0.00127	0.000633		
	Matrix 4	0.999687	1.003105	0.998815	0.003722	-0.0006	-0.00038		

The added value of all of these computations is the ability to move forward and backwards between all the coordinate systems. One of the most valuable conversions during surgery may be the desire to move from a current point to a planned point. These adjustments on the surgical head-stage need to be computed. In our sample we presume the target point (AP, LAT, VERT) coordinates to be (-3, -11.5, -3), but the current position based on intraoperative imaging is (-4.4, -10.3, -4.2). In order to move the electrode point to the target, one would need to move (0.53, -0.71, 2.02) on the head-stage (Table 4). This information is informative as one may also realize that the radial error is 0.89 mm and the Euclidean error is 2.2 mm. Therefore, the largest displacement error is along the trajectory depth.

**Table 4:** Evaluation of a planned target coordinate versus a current target coordinate. Using the information, one can evaluate the data in MidACPC space and also compute the added movement required on head-stage if a readjustment to target is desired. To go from the current position to the planned target, a movement 0.52 mm AP, LAT to left 0.71 mm, and VERT upwards 2.02 mm needs to be performed. Notably, the radial error is 0.89 mm, and the Euclidean error is 2.2 mm, which leads to the conclusion that the largest error is in the VERT dimension, but the current position is along the planned trajectory. AP = Antero-Posterior; LAT = Lateral; VERT = Vertical; MidACPC = Middle of anterior commissure and posterior commissure; CRW = Cosman-Roberts-Wells Stereotactic Frame.

	AP	LAT	VERT
CRW Planned Position	-0.8	-9.3	1.7
MidACPC Planned Position	-3	-11.5	-3
CRW Current Position	-2.36385	-8.20936	0.615163
MidACPC Current Position	-4.40882	-10.3171	-4.21997
To Get From Current to Planned Target	0.529056	-0.71043	2.021812
Radial Error from Current to Planned Target	0.88578		
Euclidean Error from Current to Planned Target	2.207336		

It may also be important to analyze how 2 mm adjustments on the head-stage effect the $midACPC$ coordinates. Here, we take the target position and make various changes on the head-stage. Each new position is seen as well as their changes to the original position in each dimension. Oftentimes, 2 mm adjustments are made by the surgeon on the head-stage, which are presented in the AP, LAT, and VERT dimension (Table 5). Finally, a new target coordinate is selected and the necessary head-stage operation to reach that coordinate from the original target is computed.

**Table 5:** AP, LAT, and VERT changes on head-stage coordinates have an effect on the MidACPC coordinates. A head-stage movement is computed by moving +2 mm AP, +2 mm LAT, +2 mm VERT on the left and right trajectories and the resulting MidACPC position is computed. The change in MidACPC from the original target is computed as delta. Finally, a new MidACPC target is chosen on the right and left side and the necessary head-stage adjustments to reach those targets are computed along with the radial error. AP = Antero-Posterior; LAT = Lateral; VERT = Vertical; MidACPC = Middle of Anterior Commissure and Posterior Commissure; mm = millimeters.

Head-Stage	AP	LAT	VERT		Delta	
Right Target	-3	11.5	-3	AP	LAT	VERT
+2 mm AP	-1.31	11.51	-4.06	1.69	0.01	-1.06
+2 mm LAT	-3.31	13.42	-3.47	-0.31	1.92	-0.47
+2 mm VERT	-1.98	12.06	-1.37	1.02	0.56	1.63
New Target	-1	11.5	-3	1.69347	-0.3106348	1.017677
					Radial Error	1.721724
	AP	LAT	VERT		Delta	
Left Target	-3	-11.5	-3	AP	LAT	VERT
+2 mm AP	-1.31	-11.49	-4.07	1.69	0.01	-1.07
+2 mm LAT	-2.75	-9.56	-2.58	0.25	1.94	0.42
+2 mm VERT	-1.96	-11.99	-1.36	1.04	-0.49	1.64
New Target	-3	-13.5	-3	-0.01461	-1.9382602	0.492883
					Radial Error	1.938315

Anatomy-Atlas Navigation (D)

**Figure 2:**
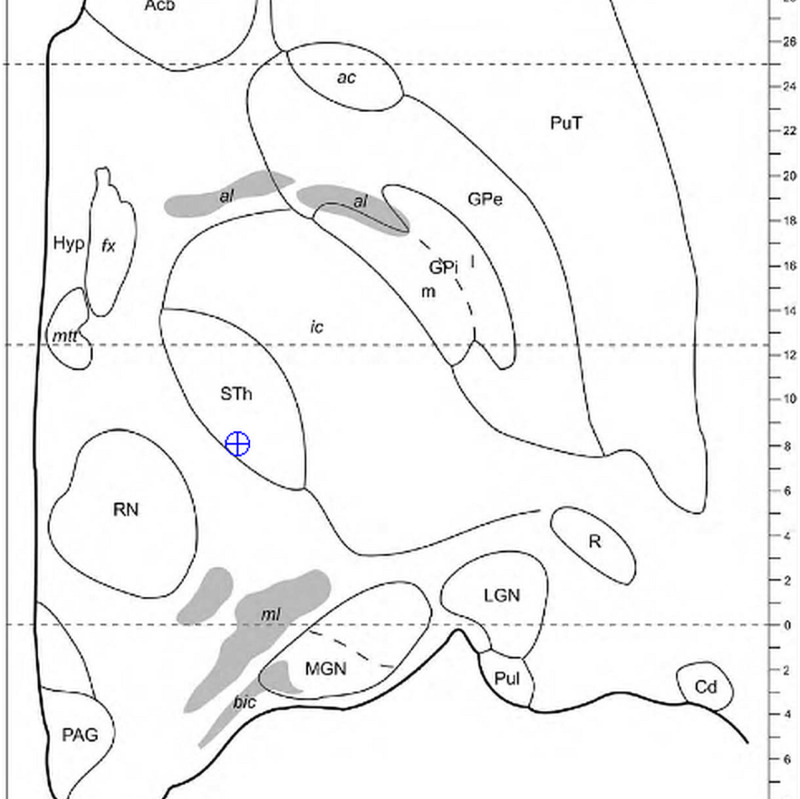
Mapping a 3D intraoperative coordinate to a point on the closest atlas section from a series. Here, we use the current point illustrated in Table 4, {-4.4, -10.3, -4.2}. Using the method, the closest axial slice of the Morel Atlas maps to {-4.4, -10.3, -4.5}. The position of the electrode tip is observed within the margins of the subthalamic nucleus (STN). All Coordinates are given as {AP, LAT, VERT} 3D = Three Dimensional Morel Atlas: please refer to reference [17].

An important aid is obtained by mapping a point to an atlas. While simple navigation of a single slice on an atlas is straightforward, we discuss the more general task of navigating all the atlas slices relative to a point. Treating the electrode position as a point ( $E$ ) and the plane as a slice from an atlas, one can then calculate the closest plane using a Point-to-Plane computation. This is done by picking three points in the plane, $P$, $Q$, and $R$. Two vectors are then created $\vec{PQ}$ and $\vec{PR}$. The normal $\hat{n}$ of the plane is then ${\vec{PQ} \times \vec{PR}}$, where $\vec{n}$ has three components, $\vec{\lambda}$, $\vec{\mu}$, $\vec{\kappa}$ and $\sigma$ can then be solved for each plane equation of the atlas sections (Equation 27). The distance $\zeta$ from point to the plane is computed, which gives the closest atlas slice (Equation 28). Then the closest point in the plane $X_{p}$ to $E$ can be computed by first solving for the unknown parameter $b$ using a known point in the plane, such as $P$ (Equation 29). Then using $b$, we can then solve for the final values $X_{p}$ (Equations 30-32). The next task is mapping $X_{p}$ to the screen coordinates. This can be accomplished using 3 pairs of coordinates of the atlas in 3D-2D (three dimensional - two dimensional) using similar mathematics previously published by Brown, where a conversion matrix $M$ is solved and then $X_{p}$ is applied to the inverse matrix to identify the screen coordinates $U_{p}$ and $V_{p}$ (Equations 33, 34) [16]. In some instances, the determinant of $M$ can be zero when using an atlas section with zeros in a dimension, making the matrix not invertible for Equation 34; in this instance, one can alternatively utilize a 2D or alternative approach. The screen coordinates are then placed on the Morel atlas (Figure 2) [17]. Finally, one can use the matrix to compute any point $X_{i}$ from any screen coordinates $U_{i}$ and $V_{i}$ (Equation 35). Notably, this same technique can be utilized with CT or MRI slices.

$$\vec{\lambda}x + \vec{\mu}y +\vec{\kappa}z + \sigma = 0\tag{27}$$

$$\zeta = \frac{\vert \vec{\lambda}E_{x} + \vec{\mu}E_{y} + \vec{\kappa} E_{z} + \sigma \vert} {(\vec{\lambda}^2 + \vec{\mu}^2 + \vec{\kappa} ^2)^{1/2}} \tag{28}$$

$$b = \frac { (P_{x}-E_{x})\vec{\lambda} + (P_{y} -E_{y})\vec{\mu} + (P_{z}-E_{z})\vec{\kappa} } { \vec{\lambda}^2 + \vec{\mu}^2 +\vec{\kappa}^2 } \tag{29}$$

$$x_{p} = E_{x} + \vec{\lambda}b \tag {30}$$

$$y_{p} = E_{y} + \vec{\mu}b \tag {31}$$

$$z_{p} = E_{z} + \vec{\kappa}b \tag {32}$$

$$\begin{bmatrix} m_{11} & m_{12} & m_{13} \\ m_{21} & m_{22} & m_{23} \\m_{31} & m_{32} & m_{33} \end{bmatrix} = \begin{bmatrix} U_{1} & V_{1} & 1 \\ U_{2} & V_{2} & 1 \\U_{3} & V_{3} & 1 \end{bmatrix}^{-1} \cdot \begin{bmatrix} x_{1} & y_{1} & z_{1} \\ x_{2} & y_{2} & z_{2} \\x_{3} & y_{3} & z_{3} \end{bmatrix} \tag{33}$$

$$\begin{bmatrix} U_{p} & V_{p} & 1 \end{bmatrix} = \begin{bmatrix} x_{p} & y_{p} & z_{p} \end{bmatrix} \cdot \begin{bmatrix} m_{11} & m_{12} & m_{13} \\ m_{21} & m_{22} & m_{23} \\m_{31} & m_{32} & m_{33} \end{bmatrix}^{-1} \tag{34}$$

$$\begin{bmatrix} x_{i} & y_{i} & z_{i} \end{bmatrix} = \begin{bmatrix} U_{i} & V_{i} & 1 \end{bmatrix} \cdot \begin{bmatrix} m_{11} & m_{12} & m_{13} \\ m_{21} & m_{22} & m_{23} \\m_{31} & m_{32} & m_{33} \end{bmatrix} \tag{35}$$

If a trajectory is known one can alternatively utilize a line-plane intersection to identify the point in a given atlas section. First, we consider the atlas section of interest, which represents a plane (Equation 27). Then the trajectory can be represented as parametric line equations (Equations 37-39). The point in the plane can then be computed with a line-plane intersection solution (Equations 40-42). Finally, the point in the atlas plane $X_{p}$ can then be mapped to the screen coordinates using Equations 33 and 34.

$$x = x_{1} + a_{1}\cdot q\tag{37}$$

$$y = y_{1} + b_{1}\cdot q\tag{38}$$

$$z = z_{1} + c_{1}\cdot q\tag{39}$$

$$x_{p} = x_{1} - \frac{a_{1}(\vec{\lambda}x_{1}+\vec{\mu}y_{1}+\vec{\kappa}z_{1}+\sigma)}{\vec{\lambda}a_{1}+\vec{\mu}b_{1}+\vec{\kappa}c_{1}}\tag{40}$$

$$y_{p} = y_{1} - \frac{b_{1}(\vec{\lambda}x_{1}+\vec{\mu}y_{1}+\vec{\kappa}z_{1}+\sigma)}{\vec{\lambda}a_{1}+\vec{\mu}b_{1}+\vec{\kappa}c_{1}}\tag{41}$$

$$z_{p} = z_{1} - \frac{c_{1}(\vec{\lambda}x_{1}+\vec{\mu}y_{1}+\vec{\kappa}z_{1}+\sigma)}{\vec{\lambda}a_{1}+\vec{\mu}b_{1}+\vec{\kappa}c_{1}}\tag{42}$$

**Figure 3:**
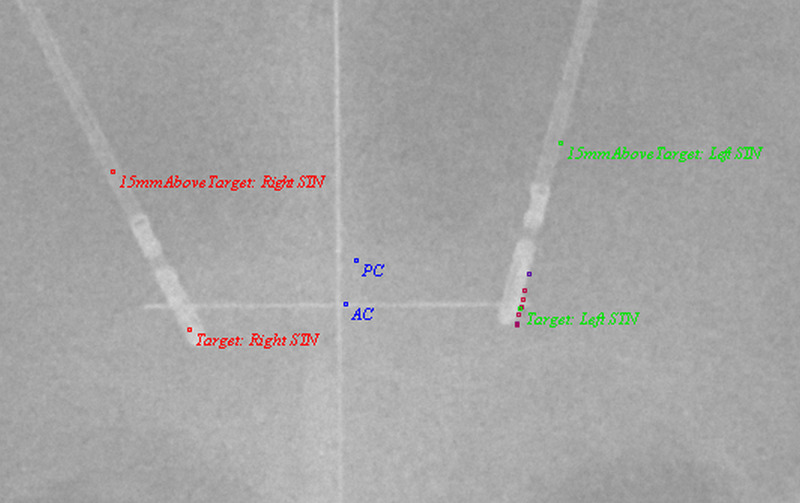
AP X-ray image showing kinesthetics acquired in 3D space and mapped to the 2D image on the left side. Note the slight disparity between MER tract, with which the kinesthetic points were acquired, and the final DBS electrode position. AP = Antero-Posterior; AC = Anterior Commissure; PC = Posterior Commissure; STN = Subthalamic Nucleus; DBS = Deep Brain Stimulation; MER = Microelectrode recording; 3D = Three Dimensional; 2D = Two Dimensional.

**Figure 4:**
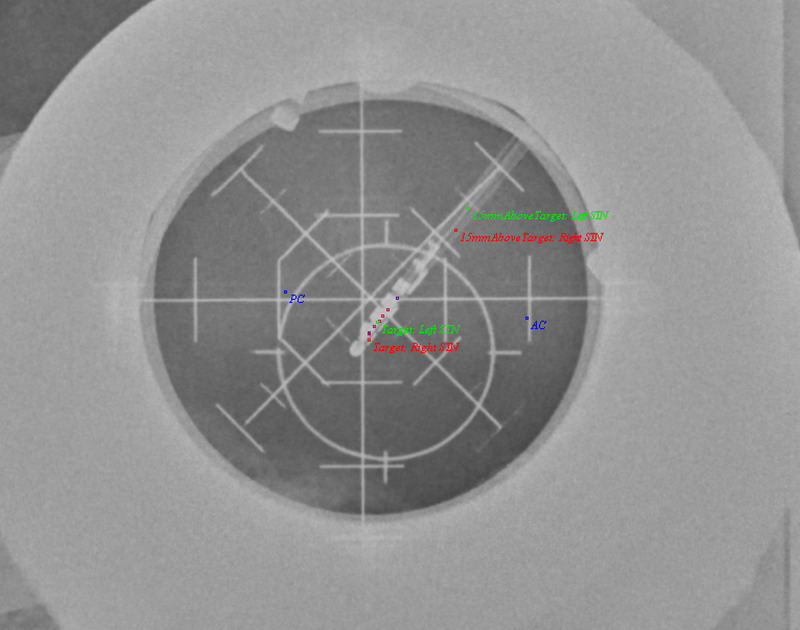
LAT X-ray image showing kinesthetics acquired in 3D space and mapped to the 2D image on the left side. Note the slight disparity between the left MER tract, with which the kinesthetic points were acquired, and the final electrode position. AP = Antero-Posterior; AC = Anterior Commissure; PC = Posterior Commissure; STN = Subthalamic Nucleus; DBS = Deep Brain Stimulation; MER = Microelectrode recording; 3D = Three Dimensional; 2D = Two Dimensional.

**Figure 5:**
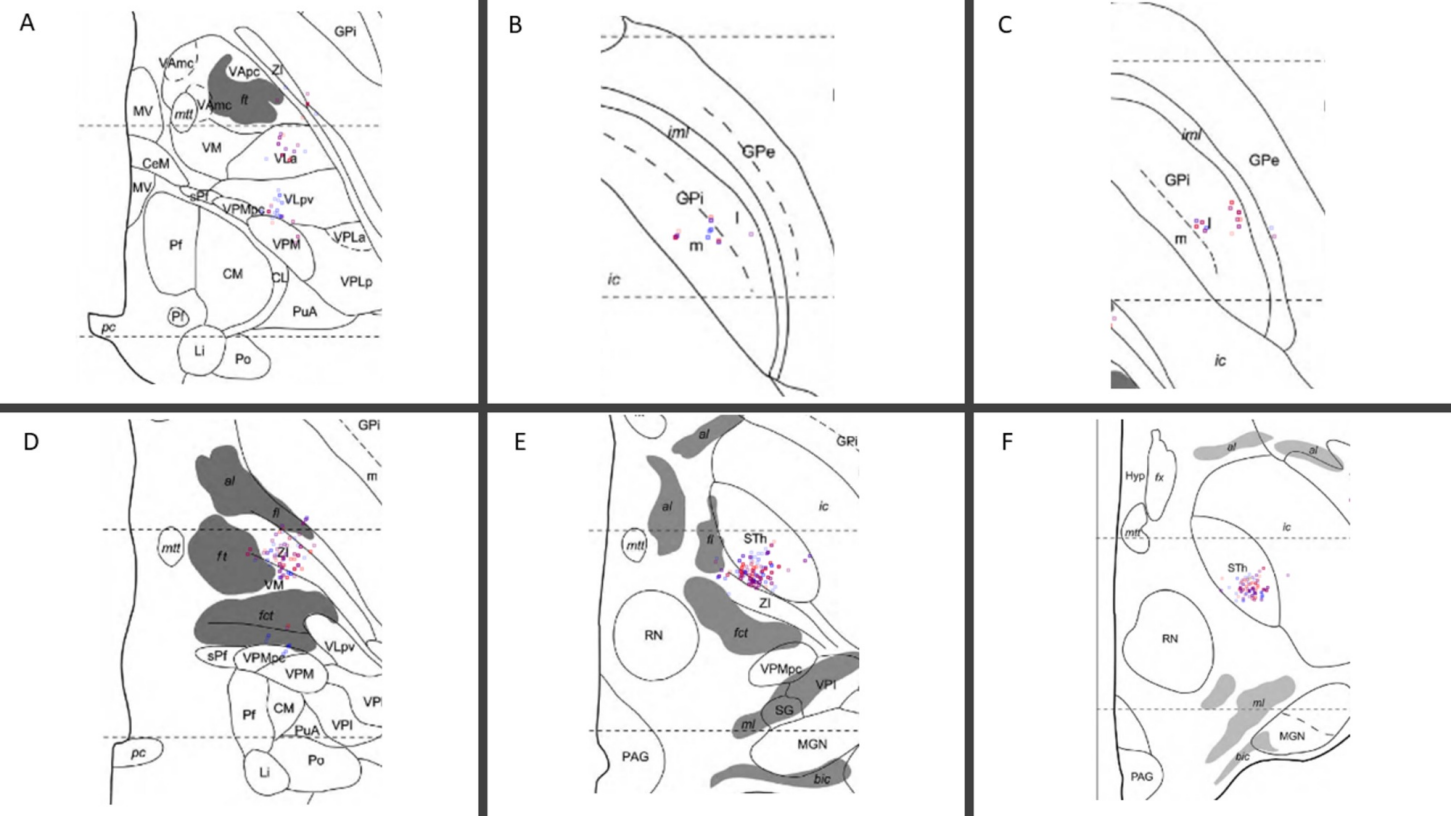
Intraoperative kinesthetic responses were computed in three-dimensional (3D) coordinates along trajectories and then mapped to points on the closest axial Morel Atlas section. These responses were computed during deep brain stimulation (DBS) targeting for ventral intermediate nucleus of the thalamus (VIM), the globus pallidus interna (GPi), and the subthalamic nucleus (STN) regions. Six of twenty-six atlas sections are displayed with the highest density of kinesthetic points acquired during VIM (A), GPi (B, C), and STN (D, E, F) targeting. Lower extremity responses are red and upper extremity responses are blue. In total, these compute to 1336 upper extremity kinesthetics, and 990 lower extremity kinesthetics. VIM = Ventral Intermediate Nucleus of the Thalamus (A) GPi = Globus Pallidus Interna (B, C) STN = Subthalamic Nucleus (D, E, F) 3D = Three Dimensional Morel Atlas: please refer to reference [17] Section A: dorsal 0.9 millimeters Section B: ventral 3.6 millimeters Section C: ventral 0.9 millimeters Section D: ventral 0.9 millimeters Section E: ventral 2.7 millimeters Section F: ventral 4.5 millimeters

Because we can now map points along a trajectory, it may be useful to compute 3D (three-dimensional) kinesthetic points, which are points acquired by along a depth during intraoperative neurophysiological mapping. Then, using stereotactic intraoperative localization (STiL), these kinesthetic points can be mapped directly to an X-ray image, which can be valuable for deep brain stimulation (DBS) programming (Figures 3, 4) [18]. These same kinesthetics can also be mapped to the atlas. Here, we have compiled 2137 individual kinesthetic points matched to the closest point on the atlas slices for the ventral intermediate nucleus of the thalamus (VIM), the globus pallidus interna (GPi), and the subthalamic nucleus (STN) (Figure 5). In VIM, one can observe that arm and leg kinesthetics appear to separate, but in GPi and STN they are largely overlapping.

**Figure 6:**
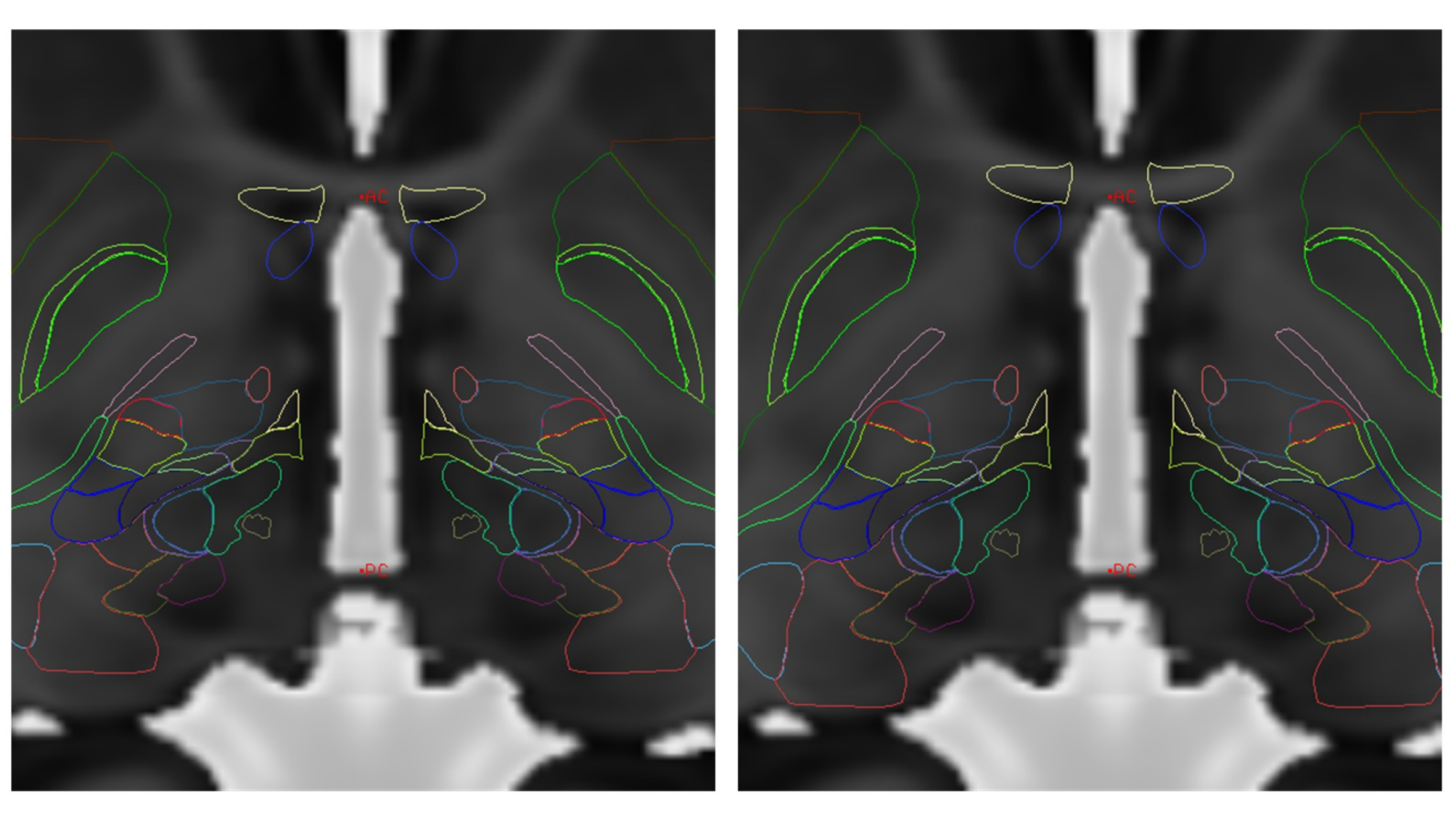
Simple scaling of AP dimension using AC-PC distance in MRI and atlas. The left image has no scaling, but is anchored on MidACPC. The right image has the AP scaling correction on this MNI Atlas over the MRI. This simple method is effective for basal ganglia targeting. MNI = Montreal Neurological Institute; AC = Anterior Commissure; PC = Posterior Commissure; MRI = Magnetic Resonance Imaging; AP = Antero-Posterior; MidACPC = Middle of Anterior Commissure and Posterior Commissure MNI Atlas = Montreal Neurological Institute Atlas, please refer to reference [19]. Figure provided by Mevis Stereotactic Planning System (MNPS)

**Figure 7:**
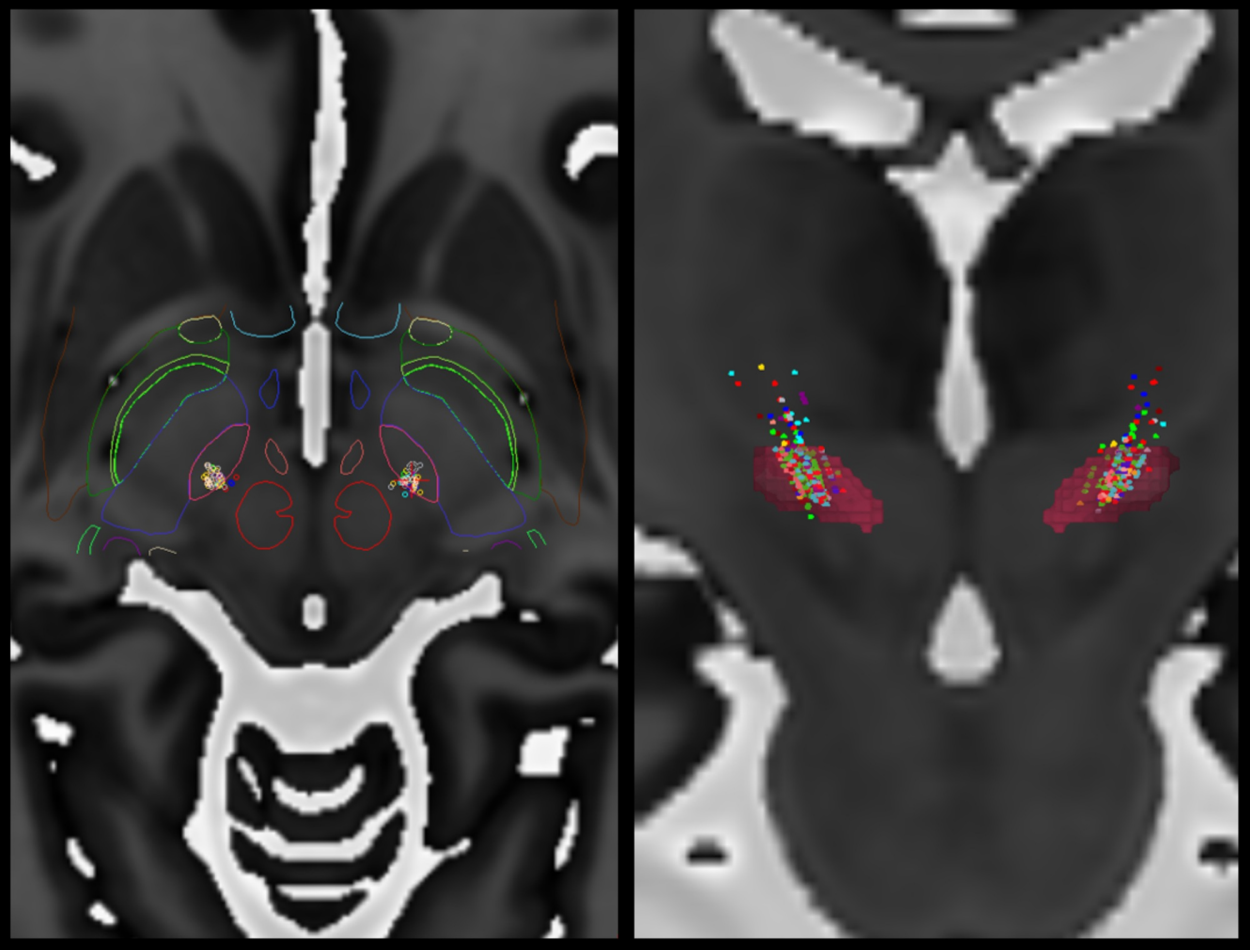
Image of 920 kinesthetic points mapped to MNI Atlas. These kinesthetic points are computed during neurophysiological testing. Axial image with superimposed outlines of subcortical structures (Left). Coronal image shows kinesthetic points along with subthalamic nucleus (red) volume rendering (Right). Note the high density of kinesthetic points in the region of the subthalamic nucleus. In this instance, color coding is arbitrary. MNI Atlas = Montreal Neurological Institute Atlas, please refer to reference [19] Figure provided by Mevis Stereotactic Planning System (MNPS)

Another approach may normalize an atlas coordinates to anatomical coordinates, or vice versa. Using the above 3PT method, information that can be used is the distance from AC to PC, which is often known in a stereotactic atlas as well. Note that for the 453-patient sample in our series, the average AC-PC distance was 25.016 mm +/- 1.42 mm. While this normalization is only in the AP axis, further sampling of comparable points on the patient MRI and the atlas may theoretically render an improved conversion matrix. While many complex atlas-to-brain normalizations exist, this method is simpler but yet effective for basal ganglia targeting. Normalization in this single AP axis provides significant improvement in registration of the Montreal Neurological Institute (MNI) atlas without any added complexity (Figure 6) [19]. Kinesthetic points can then be placed directly into these images (Figure 7).

## Discussion

Stereotactic operations in neurosurgery have become commonplace in many institutions for the treatment of movement disorders, epilepsy, and psychiatric disease. Essential to these operations is the mathematics related to Cartesian coordinate systems in Euclidean space. Stereotactic planning software generally computes target information for the surgeon, but is often insufficient for guiding decisions during the procedure. Therefore, keen understanding of coordinate systems is critical to enable the neurosurgeon to guide an electrode to the desired location. Clearly, precise targeting can affect patient outcomes [20]. For this reason, we have consolidated a complete, usable, and effective framework that navigates the various coordinate systems with examples. These methods can be implemented during surgical procedures or basic science laboratories.

Herein, we have described coordinate transformations utilized for anatomical space, frame-based space, surgical head-stage space, and atlas space. Importantly, surgical head-stage movement as it relates to the target is required by the surgeon. Further, we have demonstrated previously the ability of using simple biplanar X-ray to achieve real-time sub-millimeter localization of the position of an electrode [18]. Combining these techniques can allow a surgeon to make real-time decisions on electrode position, compute the location where important neurophysiological data are obtained, and assimilate this information into longitudinal data. These techniques may be especially important in some centers in view of the increased occurrence of asleep DBS, the reduction of intraoperative neurophysiological assessments, and the attendant omission of stimulation mapping. Since these asleep surgeries are guided purely by anatomical planning, the assurance of positioning, the comparison to prior data, and the ability to microposition (<2 mm) electrodes at the level of the head-stage may optimize the outcome. Moreover, in standard awake DBS, these techniques can also be utilized and guided by intraoperative mapping. Implementation of these principles to stereotactic neurosurgery may enhance our understanding of the normal and the pathological brain as well as the treatment of various diseases.

## Conclusions

Stereotactic neurosurgery frequently involves numerous coordinate systems. An understanding of these coordinate systems may be important for real-time surgical decisions and precise targeting. In frame-based stereotaxis, converting coordinates between different systems is important but beyond the initial stereotactic plan, these techniques are often not provided by the stereotactic planning software. Therefore, we provide a common framework with which these important techniques can be understood and implemented.

## References

[1] Rahman M, Murad G, Mocco J (2009). Early history of the stereotactic apparatus in neurosurgery. Neurosurg Focus.

[2] Compston A (2007). The structure and functions of the cerebellum examined by a new method. By Sir Victor Horsley, FRS, FRCS and R.H. Clarke, MA, MB. Brain 1908: 31; 45-124. Brain.

[3] Spiegel E, Wycis H, Marks M, Lee AJ (1947). Stereotaxic apparatus for operations on the human brain. Science.

[4] Rzesnitzek L, Hariz M, Krauss J (2019). The origins of human functional stereotaxis: a reappraisal. Stereotact Funct Neurosurg.

[5] Lasak J, Gorecki J (2009). The history of stereotactic radiosurgery and radiotherapy. Otolaryngol Clin North Am.

[6] Brown R (1979). A computerized tomography-computer graphics approach to stereotaxic localization. J Neurosurg.

[7] Brown R (1979). A stereotactic head frame for use with CT body scanners. Invest Radiol.

[8] Brown R, Nelson J (2016). The invention and early history of the N-localizer for stereotactic neurosurgery. Cureus.

[9] Cashbaugh J, Kitts C (2018). Automatic calculation of a transformation matrix between two frames. IEEE Access.

[10] Golub G, Van Loan C (1996). Matrix Computations, Third Edition. https://dl.acm.org/doi/10.5555/248979.

[11] Horn B (1987). Closed-form solution of absolute orientation using unit quaternions. J Opt Soc Am.

[12] Ott K (1998). An algorithm for the empirical determination of intracranial stereotactic targets. Stereotact Funct Neurosurg.

[13] Taub E (2000). Mathematical theory of stereotactic coordinate transformation: elimination of rotational targeting error by addition of a third reference point. J Neurosurg.

[14] Ammannati F, Bordi L, Mennonna P, Gronchi P (1999). Trigonometric method of computing the coordinates of invisible targets in functional neurosurgery. Stereotact Funct Neurosurg.

[15] Krauss J, King D, Grossman R (1998). Alignment correction algorithm for transformation of stereotactic anterior commissure/posterior commissure-based coordinates into frame coordinates for image-guided functional neurosurgery. Neurosurgery.

[16] Brown R (2015). The mathematics of four or more N-localizers for stereotactic neurosurgery. Cureus.

[17] Morel A (2007). Stereotactic Atlas of the Human Thalamus and Basal Ganglia.

[18] Sedrak M, Sabelman E, Pezeshkian P (2019). Biplanar X-ray methods for stereotactic intraoperative localization in deep brain stimulation surgery. Operative Neurosurg.

[19] Fonov V, Evans A, McKinstry R, Almli CR, Collins DL (2009). Unbiased nonlinear average age-appropriate brain templates from birth to adulthood. NeuroImage.

[20] Li Z, Zhang J, Ye Y (2016). Review on factors affecting targeting accuracy of deep brain stimulation electrode implantation between 2001 and 2015. Stereotact Funct Neurosurg.

